# Trace N-glycans including sulphated species may originate from various plasma glycoproteins and not necessarily IgG

**DOI:** 10.1038/s41467-018-05173-w

**Published:** 2018-07-25

**Authors:** Gordan Lauc, Frano Vučković, Albert Bondt, Marija Pezer, Manfred Wuhrer

**Affiliations:** 1 Faculty of Pharmacy and Biochemistry, University of Zagreb, Ante Kovacica 1, 10000 Zagreb, Croatia; 2 Genos Glycoscience Research Laboratory, Borongajska 83H, 10000 Zagreb, Croatia; 3 Center for Proteomics and Metabolomics, Leiden University Medical Centre, Zone S3, Albinusdreef 2, 2333 ZA Leiden, The Netherlands

## Introduction

In the article entitled “A method to identify trace sulphated IgG N-glycans as biomarkers for rheumatoid arthritis”, Wang et al. reported an analysis of glycans in 277 patients with rheumatoid arthritis (RA) and 141 healthy individuals^1^. The analytical method they used was very sensitive and powerful, providing results for glycans that represented only 0.005% of the glycome. They identified a total of 444 glycan structures and concluded that all these structures are N-glycans that are attached to immunoglobulin G (IgG). However, this glycomic analysis of IgG obtained by protein A purification does not account for the ubiquitous presence of varying levels of other contaminating plasma glycoproteins. As experimental evidence for association of these trace glycans with IgG was not provided, we believe that many of these glycans actually originated from other plasma glycoproteins, and not IgG.

**Fig. 1:**
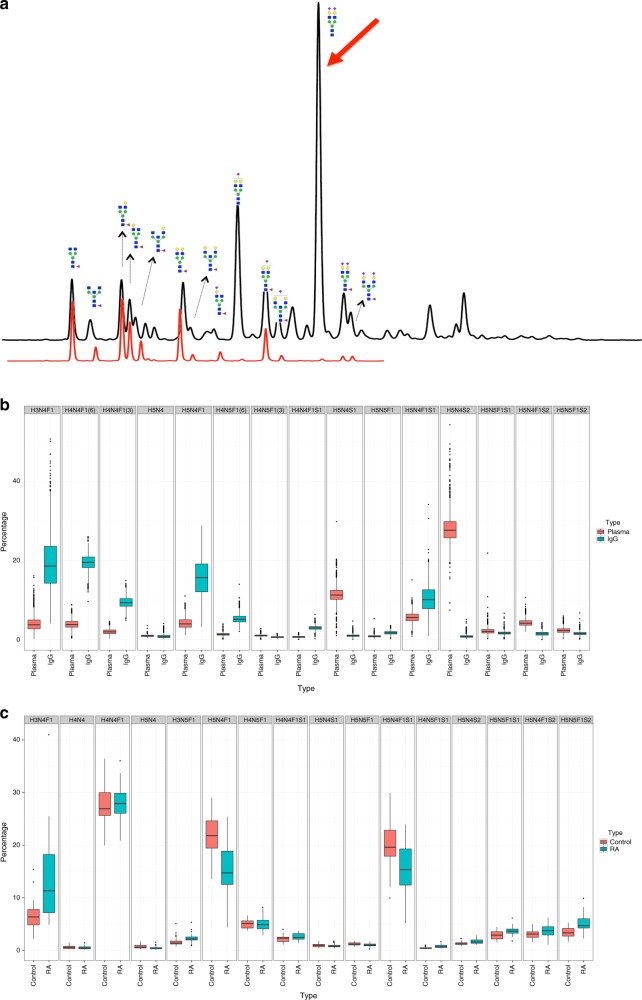
IgG and total plasma N-glycomes. **a** An overlay of UHPLC traces of IgG (red) and total plasma (black) glycomes. Major structures that are present in both total plasma and IgG glycomes are presented as cartoons composed of *N-*acetylglucosamine (blue square), mannose (green circle), galactose (yellow circle), fucose (red triangle) and sialic acid (purple diamond). The red arrow marks the largest plasma glycan peak that contains the diantennary, digalactosylated and disialylated glycan without core fucose (H5N4S2). This structure is present only in trace amounts in the total IgG glycome pool and is completely undetectable as a corresponding Fc glycopeptide^3,11,12^. **b** Composition of the total plasma and IgG N-glycomes in 2611 individuals from the general population analysed by UHPLC^5^. **c** Comparison of major IgG glycan structures in 31 patients with RA and 25 age-matched healthy individuals measured by MALDI-TOF-MS^15^

In the past few years we have analysed the glycome composition of more than 50,000 samples from different clinical and epidemiological studies^2–13^. The quality assurance measures we took enabled us to reliably determine sources of experimental variation and levels of experimental error in glycomic experiments. From these analyses we learned that one of the major sources of error in IgG glycome analysis is linked to glycoprotein contaminants. Affinity purification of IgG using proteins A or G is never absolute and varying levels of contaminating plasma proteins are always present. As most plasma proteins are glycosylated, variations in levels of contaminating proteins can have substantial effect on the apparent IgG glycome composition. For example, the diantennary, digalactosylated and disialylated glycan without core fucose (H5N4S2, or A2G2S2 in alternative nomenclature) is the most prevalent glycan on total plasma proteins, while it is present in very low amounts in the total IgG glycome (Fig. 1a–c), and practically nonexistent on IgG Fc^3,11,12^.

When released IgG glycans are analysed, even with very low levels of contaminating plasma proteins (not even visible on Coomassie-stained SDS-PAGE), the majority of H5N4S2 glycans in the “IgG glycome” actually originate from other plasma proteins such as transferrin, and not IgG^14^. Wang et al.^1^ reported that, in their study, two H5N4S2 isomers had a combined relative abundance of 5.4% (Supplementary Table 4). In our high-throughput studies of released IgG glycans (on over 30,000 individuals) this structure was less than 1% in nearly all samples. Both ultra-high performance liquid chromatography (UHPLC) and matrix-assisted laser desorption/ionization-time of flight-mass spectrometry (MALDI-TOF-MS) analyses resulted in comparable levels of this glycan (Fig. 1b, c)^6,7,15^. This structure was slightly elevated in patients with RA, mostly originating from glycans attached to Fab, but still below 1.5% of the total IgG glycome (Fig. 1c).

We consider that high levels of H5N4S2 are an indicator of contamination with other glycoproteins and use it for quality assessment of IgG purification. The fact that the H5N4S2 glycan represents over 5% of the IgG glycome in the study of Wang et al.^1^ indicates that the purified IgG may contain substantial levels of contaminating plasma glycoproteins. Furthermore, this glycan had a particularly high standard deviation, again suggesting that it was mainly derived from varying levels of contaminating plasma glycoproteins. The study by Wang et al.^1^ may therefore be confounded by sample purity issues, and the claim that the novel glycans are linked to IgG seems insufficiently supported. To the contrary, many of these low-abundance glycan structures may be derived from contaminating plasma proteins, and not or only to a lesser extent from IgG.

Figure 3 of Wang et al.^1^ implies that the novel glycan structures are actually Fc glycans, which may be questioned as the human IgG glycome is composed of glycans from the Fc as well as the Fab portion of the molecule. A state-of-the-art method of confirming IgG as the carrier molecule of the glycans and revealing the attachment site in the Fc portion of the molecule would be a complementary glycopeptide analysis. In the study by Wang et al.^1^ this was not performed and therefore the claim that IgG is the carrier of the 444 different N-glycan structures is not substantiated with experimental evidence.

We do not imply that any of the experimental data presented in the manuscript are false, or that association with rheumatoid arthritis is unsubstantiated. However, we challenge the conclusion that the analysed glycans originate from IgG, as this claim is not substantiated by the experimental evidence.

## Methods

UHPLC analysis of IgG and total plasma N-glycome was performed on 2611 individuals as described previously^5,6^. Briefly, IgG was isolated by affinity chromatography on protein G. N-glycans from isolated IgG and from plasma samples were released with PNGase F, labelled with 2-aminobenzamide and analysed by UHPLC. IgG N-glycan chromatograms were separated into 24 and total plasma N-glycan chromatograms into 39 glycan peaks. The content of each glycan peak had been determined previously by MS and exoglycosidase digestion.

MS analysis of IgG N-glycome in 31 patients with RA and 25 age-matched healthy individuals was performed as described previously^15^. Briefly, IgG was isolated by affinity chromatography on CaptureSelect IgG-Fc (Hu) beads. N-glycans from isolated IgG were released with PNGase F, ethyl esterification was performed to prevent the loss of sialic acids, and released glycans were analysed by MALDI-TOF-MS.

### Data availability

All relevant data are available from the corresponding author upon reasonable request.
